# Moyamoya as a Cause of Altered Mental Status in the Emergency Department: A Case Report

**DOI:** 10.5811/cpcem.2020.10.49415

**Published:** 2021-01-22

**Authors:** Michael West, Elizabeth Dearing

**Affiliations:** The George Washington University Hospital, Department of Emergency Medicine, Washington, District of Columbia

**Keywords:** Seizure, stroke, diagnostic bias

## Abstract

**Introduction:**

This case reviews a patient with moyamoya disease, a rare cause of altered mental status. It highlights the importance of maintaining clinical suspicion for uncommon causes of common presentations.

**Case Report:**

A 64-year-old male presented with seizures and persistent altered mental status. Computed tomography demonstrated findings consistent with bilateral ischemia. Cerebral angiography was performed with no thrombus identified but moyamoya disease present.

**Conclusion:**

Although rare, moyamoya should be considered as a potential cause of patients presenting with altered mental status. The case presented also highlights the importance of avoiding common diagnostic biases.

## INTRODUCTION

Altered mental status is a common presentation to the emergency department (ED), accounting for between 1–10% of all encounters.^1^ While some presentations of altered mental status have a clear etiology based on provided history, vital signs or examination, many patients present undifferentiated and, therefore, require a broad evaluation including laboratory testing and neuroimaging. This case reviews a patient presenting with moyamoya disease, a rare cause of altered mental status, and highlights the importance of maintaining clinical suspicion for uncommon causes of common presentations.

## CASE REPORT

A 64-year-old Black male with a reported history of seizure disorder was brought to the ED by emergency medical services (EMS) for altered mental status. Prior to arrival to the ED the patient was found by paramedics with seizure activity, and midazolam 5 milligrams was administered intravenously, causing cessation of the tonic-clonic movements. The patient remained unresponsive during transport and was not intubated prior to arrival. He was altered on arrival to the ED. There was no family present with patient, no family contact information available, and no prior documentation in the electronic health record; therefore, no additional history was available.

The patient arrived nonverbal with minimal spontaneous movements of all extremities and was not consistently following commands. He was protecting his airway and maintained adequate oxygen saturations. Given these findings on arrival and that the patient was likely in a postictal period without sign of ongoing seizure and improvement in his Glasgow Coma Scale compared to the EMS report, the physicians decided to not intubate the patient on arrival and closely monitor his neurologic status. Initial vitals also revealed that he was afebrile but with an initial blood pressure of 84/52 millimeters of mercury (mmHg). A point-of-care ultrasound was performed to evaluate the hypotension and revealed no sites of bleeding and normal cardiac output. Intravenous fluids were administered and blood pressure quickly normalized. Electrocardiogram was unremarkable. On repeat neurological examinations, the patient continued to exhibit altered mental status, clinically inconsistent with a postictal state.

Laboratory workup revealed leukocytosis to 14.5 × 10^3^ cells per microliter (mcL) (reference range 4.80–10.80^3^ mcL), as well as a lactate of 9.3 millimoles per liter (mmol/L) (reference value for critical high of > 4.0 mmol/L). Venous blood gas was significant for a pH of 7.25 (reference range 7.32–7.42) with a partial pressure of carbon dioxide of 46.6 mm Hg (reference range 25–40 mm Hg) and bicarbonate level of 20 mmol/L (reference range 24–28 mmol/L). The patient was given vancomycin and piperacillin/tazobactam as empiric antibiotics for potential infectious cause as well as levetiracetam.

A computed tomography (CT) of the brain without contrast showed loss of gray/white matter differentiation on the right side, evidence of right middle cerebral artery (MCA) acute infarction. The on-call neurologist was emergently consulted. The patient was not eligible for tissue plasminogen activator. CT angiography with perfusion imaging was then emergently pursued and showed bilateral MCA infarction at the first segment (M1) with core to penumbra mismatches and no significant core seen on the left. Based on this imaging, the neurology team activated the large cerebral vessel occlusion protocol. The patient was subsequently taken for cerebral angiography, which revealed bilateral internal carotid artery stenosis. Additionally, it found evidence of stenosis of the right and left M1 sections but no thrombus. Extensive perforator collateral circulation was found, consistent with moyamoya disease (Image). No additional intervention was indicated, and the patient was admitted to the intensive care unit. He was eventually discharged to a rehabilitation facility with residual left-sided hemiparesis and dysphagia.

## DISCUSSION

The differential diagnoses and, therefore, the evaluation for a patient presenting with altered mental status is broad. Seizures are one potential cause of altered mental status and a common complaint in the ED, accounting for 1–2% of ED visits annually.^2^,^3^ Although patients may be unresponsive or obtunded immediately following a seizure, they should show gradual improvement in their mental status. If a patient is not improving clinically or there are findings inconsistent with seizure, alternative causes must be explored.

**CPC-EM Capsule**

What do we already know about this clinical entity?

Moyamoya, a rare disease with progressive stenosis of the internal carotid arteries, is associated with strokes and seizures in children and adults.

What makes this presentation of disease reportable?

While moyamoya is classically found in those of Asian descent, we describe a complex presentation of a Black adult male with generalized symptoms of altered mental status.

What is the major learning point?

This case highlights the diagnosis of moyamoya disease as a rare cause of altered mental status while emphasizing the need to avoid bias in clinical decision-making.

How might this improve emergency medicine practice?

Crucial to safe, accurate diagnosis is continued diagnostic suspicion in atypical presentations and consideration of rare causes of common presentations.

Moyamoya, meaning “a puff of smoke” in Japanese, is a rare disease typically affecting the internal carotid arteries in which the lumens of those vessels progressively narrow due to smooth muscle proliferation.^4^,^5^ Over time, collateralization occurs; however, the lack of carotid vessel flow places the patient at higher risk of transient ischemic attacks, arteriovenous malformations, and stroke.^6^,^8^ Classically, this disease has been described in Asia and is often found in childhood. Moyamoya can also present in adulthood as headaches, seizures, altered mental status, transient ischemic attack, and stroke.^7^ Presentations in the United States tend to be later in life and are generally associated with a lower stroke recurrence rate and better functional outcomes due to a more robust collateral circulation.^9^,^10^

In this case, the finding of seizure activity confirmed by paramedics could have potentially led to confirmation bias given the patient’s reported history of epilepsy. Elevation in lactate is common in patients after a seizure; however, the hypotension on arrival in combination with an elevated lactate could have also led the physician to anchor on infection as the cause. Additionally, the findings on neuroimaging did not correlate directly to the patient’s initial neurological exam findings. Finally, the radiologist theorized that heart failure could potentially have been the cause of the inadequate contrast load to the brain and subsequent bi-hemispheric findings; however, this was confounded by point-of-care transthoracic ultrasonography showing sufficient cardiac function.

## CONCLUSION

By maintaining clinical suspicion for alternate diagnoses as well as pursing continued diagnostic testing and patient reassessment, the rare finding of moyamoya disease, was identified. Moyamoya could be considered in adult patients presenting with acute seizures or strokes in addition to patients with altered mental status.

## Figures and Tables

**Image:**
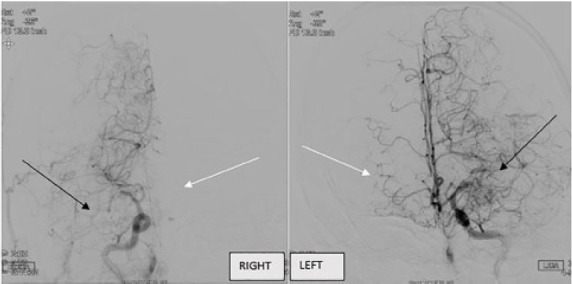
Collateralization of the cerebral vessels on cerebral angiography. The collateralization, and therefore cerebral blood flow, on the left is more extensive than the right, consistent with left-sided deficits. Differences in cerebral blood flow can be visualized by comparing black arrows and white arrows.
